# Interactions of *Metarhizium brunneum*-7 with Phytophagous Mites Following Different Application Strategies

**DOI:** 10.3390/insects11060330

**Published:** 2020-05-28

**Authors:** Dana Ment, Sukirtha Raman, Shira Gal, David Ezra, Eric Palevsky

**Affiliations:** 1 Department of Entomology and Nematology, Plant Protection Institute, Agricultural Research Organization (ARO), The Volcani Center, HaMaccabim Road 68, Rishon LeZion 7528809, Israel; sukirtharaman@gmail.com; 2 Department of Entomology and Nematology, Plant Protection Institute, ARO, Newe Yaar Reseach Center, P.O. Box 1021, Ramat Yishay 30095, Israel; shiragal@volcani.agri.gov.il (S.G.); palevsky@volcani.agri.gov.il (E.P.); 3 Department of Plant Pathology and Weed Research, Plant Protection Institute, Agricultural Research Organization (ARO), The Volcani Center, HaMaccabim Road 68, Rishon LeZion 7528809, Israel; dezra@volcani.agri.gov.il

**Keywords:** Microbial control, entomopathogenic fungus, Hypocreales, live confocal imaging, *Rhizoglyphus robini*, *Phyllocoptruta oleivora*, *Tetranychus urticae*, spray application, drench application

## Abstract

*Metarhizium brunneum* is a generalist entomopathogenic fungus known to be virulent against Acari. We investigated *Metarhizium* *brunneum*-7 (Mb7) interactions in three systems of phytophagous mites and their respective plant hosts: Volkamer lemon (*Citrus volkameriana*) and the citrus rust mite *Phyllocoptruta oleivora*; common bean (*Phaseolus vulgaris*) and the two-spotted spider mite *Tetranychus urticae*; and spring onion (*Allium cepa*) and the bulb mite *Rhizoglyphus robini*. All three mite species were susceptible to directly applied Mb7 conidia. Results obtained using the standard method for studying endophytic colonization vs. live confocal imaging of plant tissues using the green fluorescent protein (GFP)-transformed fungus differed markedly, demonstrating that microscopy validation was more definite than the standard process of recovery from plant tissue. Endophytic colonization was observed in conidium-infiltrated citrus leaves and in roots of onion plants treated with soil-drenched conidia, but not in common bean treated by either spray or drench of conidia. Endophytic colonization of citrus leaves did not affect the citrus mite population. Drench application in common bean reduced two-spotted mite population. Similarly, drench application in onion reduced bulb mite population. This study emphasizes the importance of the host plant effects on Mb7 control efficacy of mite pests, and the merits of live-imaging techniques in studying endophytic interaction.

## 1. Introduction

For decades the worldwide strategy for pestiferous mite (Acari) control has relied mainly on synthetic acaricides (organophosphates, carbamates, pyrethroids, and macrocyclic lactone [1]), and these precluded the use of alternative strategies such as biocontrol agents like predatory mites and insects [2,3,4]. As many conventional mite products are being withdrawn from markets, banned, or are no longer effective due to resistance of the mites, these and other biocontrol agents are now becoming more relevant [1,5,6]. 

Entomopathogenic fungi belonging to the order Hypocreales play central roles in the control of pestiferous mite populations [7]. Entomopathogenic fungi are typically applied repeatedly, ensuring a high number of infective units aimed at suppressing the pest population. However, the fungal conidia are highly sensitive to environmental conditions, such as UV radiation, and have to be properly dispersed and fixed in the plant canopy to demonstrate significant pest-control efficiency [8,9,10,11]. Persistence of entomopathogenic fungi in the environment depends on habitat architecture, host behavior, and environmental factors. In the soil, *Metarhizium* spp. propagules can persist for several years [12,13]. In the crop canopy, persistence was only 16–28 days for *Beauveria bassiana* conidia [12,14,15] and 3 days for *M. anisopliae* sprayed on corn [16]. In recent years, a number of studies have demonstrated the establishment of *Metarhizium* spp. in various associations with plants, resulting in a secondary effect on successive generations of pest populations [17,18,19]. Two of the major associations studied were rhizosphere competence and endophytic interactions of entomopathogenic fungi with various plants [20,21]. Both of these associations play an ecological role in diverse plant–pest and plant–pathogen systems, protecting plants against insects, mites, and pathogens, and have mostly a positive effect on plant growth [18,19,22]. 

Fungal endophytes are defined as plant-inhabiting fungi that, at some time during their lives, colonize internal tissues without causing apparent harm to their hosts [23]. Endophytes have a variety of positive effects on different aspects of their hosts’ ecological fitness, such as growth enhancement and increased tolerance to biotic and abiotic stresses [24]. Some endophytes are known to induce resistance of their host by priming of the host defense system, while others are secreting secondary metabolites that influence pathogens in a direct or indirect way as antibiosis or induce defense accordingly. In other cases, the endophytes are producing plant hormones or fixing nitrogen that promotes plant growth. Endophytes are known to improve plant resistance to drought, salinity, and other abiotic stresses. However, the mode of action is not known in all cases, but in some it is assumed that there is involvement of secondary metabolites secretion and plant defense system priming [24]. Endophytic entomopathogenic fungi, such as *Metarhizium* spp. and *Beauveria* spp., are known to inhibit the growth and reproduction of a wide range of herbivores from different feeding guilds. These include members of the Coleoptera, Diptera, Lepidoptera, and Orthoptera [18,25,26,27]. 

As numerous species of phytophagous mites infest plants of economic importance worldwide [4], sustainable solutions for mite control are highly needed. Still, only a few studies have been conducted on the effects of the method of entomopathogenic fungi conidia application and endophytic establishment on phytophagous mite populations [22,28]. We hypothesized that: (i) foliar spray and root drench application of *M. brunneum*-7 (Mb7) will result in localized and systemic colonization of plant tissues, respectively; (ii) endophytic or rhizospheric colonization of plants with Mb7 will affect mite populations; (iii) mites that are susceptible to Mb7 through direct contact with its conidia will also be affected by feeding on Mb7-colonized plant tissues. We assessed the hypotheses by studying the interactions of *Metarhizium brunneum* isolate Mb7 in three plant systems that include different phytophagous mites: (i) Volkamer lemon (*Citrus volkameriana*) and the citrus rust mite *Phyllocoptruta oleivora* (Ashmead); (ii) common bean (*Phaseolus vulgaris*) and the two-spotted spider mite *Tetranychus urticae* Koch; and (iii) spring onion (*Allium cepa*) and the bulb mite *Rhizoglyphus robini* Claparede. In each fungus–mite system, the effect of Mb7 application (spray, infiltration, or drench) on the studied mites was investigated. The study objectives were to: (i) localize and describe Mb7 colonization of the studied plants following application of conidia, and (ii) determine the efficacy of Mb7 for mite suppression when applied directly vs. when present as an endophyte or in the rhizosphere of the host plant.

## 2. Materials and Methods 

### 2.1. Mites

Citrus rust mites (*Phyllocoptruta oleivora*) were obtained from the Israel Cohen Institute for Biological Control, Bet Dagan, Israel, where they were routinely maintained on citrus seedlings placed in climate-controlled chambers (24 ± 2 °C and 60 ± 10% relative humidity (RH)). Two-spotted spider mites (*Tetranychus urticae*) were obtained from BioBee, Israel (https://www.biobee.com/), where they were grown on bean plants. Bulb mites (*Rhizoglyphus robini*) were collected from an infested onion field in Beit-Alfa, Israel (32°30′58″ N 35°25′49″ E) during the fall of 2016 and reared at the Newe Yaar Research Center on peanut paste on moist filter paper in Petri dishes, which were kept in the dark at 25 °C.

### 2.2. Fungal Strain

Mb7 constitutively expressing green fluorescent protein (GFP) [29] was grown on Sabouraud dextrose agar (SDA; Difco, BD, USA) for two weeks at 28 °C and its conidia were harvested by scraping the agar and suspending the scrapings in sterile, distilled water containing 0.01% (v/v) Triton X-100 in glass tubes. The suspension was vortexed and filtered through Miracloth (Calbiochem, La Jolla, CA, USA), and spore concentrations were determined with a hemocytometer. Suspensions were adjusted to the required conidial concentrations in 0.01% Triton X-100 and the percentage of viable conidia was determined on SDA prior to each bioassay. Only conidial suspensions with at least 95% germination were used. 

### 2.3. Plant Material

Six-month-old *C. volkameriana* seedlings in 500 mL pots were watered daily to soil moisture capacitance. 

Organic seeds of common bean (*P. vulgaris*) were purchased from an organic store. They were surface-sterilized in 70% ethanol for 1 min, washed with a solution of 3% (v/v) sodium hypochlorite in sterile distilled water for 1 min, and washed again with sterile distilled water. Seeds were dried on sterile filter paper and placed on moist cotton wool. Once germinated, seedlings were transferred to 200 mL pots containing sterile vermiculite and watered twice a week. For the experiment, we used plants that were 30 cm in height and had about 20 leaves.

Top sets (bulblets) of the onion cultivar ‘Ori’ were sterilized after removing a thin layer of tissue with forceps and the sets were washed several times. First, they were washed with water and soap, then soaked in 70% ethanol for 20 s, followed by sterile distilled water, and finally soaked in 0.2% (v/v) of the fungicide mercaptan for 15 min. The bulblets were washed again with sterile distilled water following with 3% sodium hypochlorite for 15 min, followed by a final wash in sterile distilled water. The sets were dried on sterile filter paper and planted in plastic cells containing sterile vermiculite. 

All plants were kept in a closed greenhouse with constant conditions of 25 °C and a 14:10 h light:dark regime.

### 2.4. Fungal Inoculation of Plant Material

All host plants were single time inoculated with a conidial suspension of Mb7 (10^7^ conidia mL^−1^). The target sites for inoculation in each plant host and the inoculation techniques used are summarized in Table 1. The three different inoculation techniques (foliar spray, leaf infiltration, and soil drench) depended on the host plant and the location of the mites. Bean plants were inoculated by spraying, since infiltration into the leaves was destructive, or by soil drenches. Onions were treated by drenching, since the bulb mite resides only in the below-soil plant tissue. Due to the herbaceous nature of the bean and onion plants, inoculation by drenching was designed to determine whether the fungus can spread to other plant tissues through the root system. The citrus plants were not drenched. 

The soil drench treatment was carried out by pouring a conidial suspension on the medium surface, close to the stem, at a volume of 50 mL for bean plants and 10 mL for onion bulblets. All control plants were treated with sterile distilled water with 0.01% Triton X-100.

For the spray application, plants were inoculated individually with a manual hand sprayer, while the top part of the pot was covered with aluminum foil to prevent any runoff of fungal conidia into the soil. After spraying, plants were covered with plastic bags for 24 h to maintain high humidity and enhance conidial germination, as described in Parsa et al. [30]. Citrus plants were sprayed with 20 mL conidial suspension and common bean plants were sprayed with 10 mL conidial suspension to obtain full coverage of the foliage. For inoculation by leaf infiltration, all of the leaves of each citrus plant were treated with 0.1 mL of Mb7 conidial suspension, infiltrated to the leaf by a syringe without needle. The tip of the syringe was pressed against the underside of the citrus leaves while applying gentle pressure to the other side of the leaf. 

### 2.5. Plant Sampling for Assessment of Endophytic Colonization and Live Imaging

Sampling times of the plant tissues differed among the different host species due to differences in conidial development during the first 24 h after treatment. Infiltrated citrus leaves were sampled 5 h post infiltration, and once a week for nine weeks. Bean leaves were sampled 30 min after spraying, as soon as the leaves dried, and then every five days up to 14 days post-treatment. Onion roots were sampled by carefully removing the plants from the pots and cutting off pieces of root tissue with sterile scissors. After sampling, the plants were replanted in their pots. Roots were sampled 1 h after drench application and once daily for five days. On each sampling date, leaves were sampled from six different host plants, six randomly selected leaves from each plant (both treated and controls).

Two methods were used to evaluate endophytic colonization: (i) surface sterilization and plating on selective media and (ii) live imaging by confocal laser-scanning microscopy (CLSM). The first method was used as described by Behie et al. [31], with slight modifications. The selective medium used in this study was SDA supplemented with 0.2 g L^−1^ chloramphenicol and 0.1 g L^−1^ Dodin (Pestanal, Sigma). Surface-sterilized plant parts, 4–5 per Petri dish, were incubated on the selective medium in the dark at 28 °C until the fungal colony sporulated, which took about four weeks for all samples. Plates with visible colony formation were inspected under a stereomicroscope (SMZ1270, Nikon) and samples of sporulating colonies were observed under a light microscope (E200, Nikon), to confirm the morphology of *Metarhizium* spp. conidiophores [32]. The second method, live imaging by CLSM, was performed as previously described [29]. The sampled tissue was cut with a sterile scalpel to fit a glass microscope slide. Samples were observed by CLSM within 1–2 h of being collected. For each type of sample from every treatment, at least 10 different plant material or mite preparations were observed. Selected pictures represent all pictures taken for any given sample type. 

### 2.6. Leaf Disc Assay for P. oleivora Susceptibility to Mb7 

This assay was conducted to determine whether *P. oleivora* is susceptible to Mb7 following direct application of conidia to citrus leaf discs and to leaf discs endophytically infested with the fungus. Treatments included untreated leaf discs (control), leaf discs that had been immersed for 20 s in an Mb7 suspension (1 × 10^7^ conidia mL^−1^), and leaf discs cut from leaves one week after they were infiltrated with Mb7. Leaf discs were set on polyacrylamide gel in 55 mm Petri dishes. *Phyllocoptruta oleivora* were placed on leaf discs transferred with a camel-hair brush, counted and incubated at 25 °C and ~85% RH under a 12:12 h light:dark regime. Numbers of infected mites (i.e., mites on which mycelia developed) were counted three and four days after inoculation under a stereomicroscope (SMZ1270, Nikon). Each treatment was replicated three to four times, 10–30 mites per replicate. The whole bioassay was repeated three times.

### 2.7. In-Vitro Assay for R. robini Susceptibility to Mb7

Sterile filter paper was placed in 55 mm diameter Petri dishes and impregnated with 0.5 mL sterile water containing 0.01% Triton X-100 without (control) or with fungal conidia at 1 × 10^7^ conidia mL^−1^ (i.e., 5 × 10^5^ conidia cm^−2^). Adult mites (20–30 per dish) were placed on the impregnated filter paper with a camel-hair brush, counted and incubated at 28 °C and ~85% RH in the dark. The number of infected mites (i.e., mites on which mycelia developed) was counted daily under a stereomicroscope (SMZ1270, Nikon). Each treatment was replicated five times.

### 2.8. In-Planta Bioassay for T. urticae susceptibility to Mb7 

These trials were conducted to determine the efficacy of Mb7 for mite suppression when applied directly on the foliage vs. application by drenching the root with conidial suspension. The bioassays included plants that had been drenched or sprayed with Mb7 (Section 2.4), and untreated control plants. Twenty-five adult *T. urticae* females (five per leaf) were transferred to bean plants with a camel-hair brush. These mites were treated with spraying of a conidial suspension of Mb7 (1 × 10^7^ conidia mL^−1^) to full coverage of both sides of the leaf. This was after the plants had been either sprayed or drenched with Mb7. Plants were incubated at 25 °C under a 12:12 h light:dark regime. One week later, the mites were counted on the whole plants. Each treatment was replicated six times, with each plant serving as one replicate. The experiment was repeated three times, independently, with new fungal material, plant material and mite populations.

### 2.9. In-Planta Bioassay for R. robini Susceptibility to Mb7

These trials were conducted to determine the efficacy of Mb7 in preventing mite infestation of the roots. Experiments with onion bulblets were initially conducted with onions obtained from *Fusarium*-infected soils. The second and third experiments were repeated with bulblets from Hazera which were surface sterilized as described in Section 2.3. In the first experiment, onions from *Fusarium*-infected soil were planted in pots with 800 g potting mix, watered from the bottom, incubated at 25 °C, and kept under a 16:8 h light:dark regime. In the second and third experiments, the bulblets were thoroughly cleaned and planted, two per pot. They were then drenched with 10 mL of Mb7 conidial suspension (10^7^ conidia mL^−1^). After two days, each onion plant was infested by placing 20 mites adjacent to the bulblet, for a total of 40 mites per pot. The experiment consisted of six pots with onion and bulb mites (control) and six pot with onion, bulb mites and Mb7 (treatment). Onion roots were sampled three days after planting for live imaging. After four weeks (two–three generations of bulb mites), the onions were pulled up and the mites were extracted from the bulblets in Berlese funnels for counting. Bulb diameter (cm), plant height (cm), and fresh plant weight (g) were recorded. The experiment was repeated three times, independently, with new fungal material, plant material and mite populations.

### 2.10. Data Analysis

All statistical analyses were performed using JMP^®^ Version 14 (1989–2019) software (SAS Institute Inc., Cary, NC, USA). Results are presented as mean ± standard error (SE) of replicate analyses and are either representative of or include at least three independent experiments. Means of replicates were subjected to statistical analysis and considered significant when *p* ≤ 0.05.

The number of live mites was counted on each counting day and the percentage of mite survival was calculated. The percentage of surviving mites was arcsine-square-root-transformed and subjected to repeated-measures analysis of variance (ANOVA). If differences among treatment means were found to be significant (*p* < 0.05), a *t*-test or Tukey’s test was used for comparisons among means for the leaf disc assay with *P. oleivora*, Petri dish assay with *R. robini*, and in-planta assay with *T. urticae* and *R. robini* for the designated count day. 

The mean percentages of survival in each control and Mb7-treated group were submitted to the Probit test with inverse prediction probability of 0.5 to find the predicted lethal time value for which survival is 50%, referred to as LT_50_.

The number of plant samples colonized with Mb7, either endophytically or in the rhizosphere, was counted out of the total number of samples observed. Percentage colonization was calculated based on these data. Onion plant growth data were subjected to ANOVA. If differences among the treatment means were significant, Tukey’s test was used for multiple comparisons among means. 

## 3. Results

### 3.1. Assessment of Colonization in Plants Inoculated with Mb7 by Re-Isolation and Live Imaging

**Figure 1:**
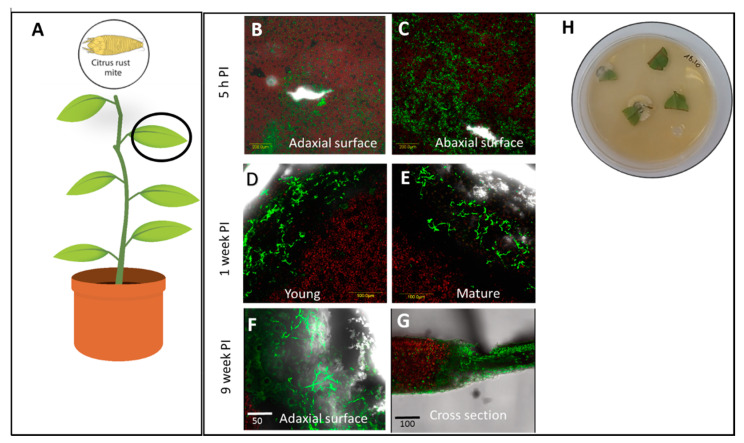
Observation of endophytic establishment of *Metarhizium brunneum*-7 (Mb7) in citrus leaves following leaf infiltration with 0.1 mL conidia suspension (10^7^ conidia ML^−1^) at three time points post inoculation. (**A**) Site of infiltration and examination of citrus seedlings. (**B**–**G**) Leaf colonization by Mb7 by confocal laser scanning microscopy (CLSM). (**H**) Recovery of endophytic Mb7 from leaf tissue fragments. PI, post inoculation. Green florescent, Mb7-green fluorescent protein (GFP); red fluorescent, plant tissue.

Plant tissue colonization was confirmed by two methods: Live imaging by CLSM and recovery from plant tissues. The colonization and endophytic establishment of Mb7 depended on the plant host and the inoculation method (Table 1). In citrus leaves, while all samples treated by infiltration were colonized, as seen by CLSM, attempts to recover the fungus from the tissues resulted in 50.0 ± 12.3% recovery (Figure 1H). Infiltration of citrus leaves (Figure 1A) resulted in endophytic colonization near the site of infiltration, up to 400 µm into the tissue, and that colonization was stable for at least nine weeks (Figure 1B–G).

**Figure 2:**
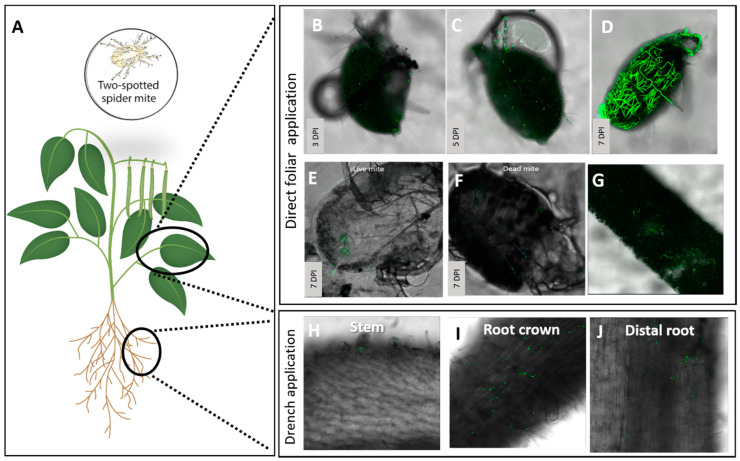
Observation of *Tetranychus urticae* mite infestation and endophytic establishment of Mb7 in bean plants following spray and drench application with conidia suspension (10^7^ conidia mL^−1^). (**A**) Sites of spray, drench and examination of bean plants. (**B**–**D**) Progression of Mb7 infection in adult *T. urticae* mites after direct application on plant. (**E**,**F**) Adult mites’ acquisition of Mb7 conidia from the leaf surface seven days after a spray application. (**G**) Mb7 conidia on a bean leaf cross section. (**H**–**J**) Mb7 conidia and germlings on bean roots after a drench application. DPI, days post inoculation.

**Figure 3:**
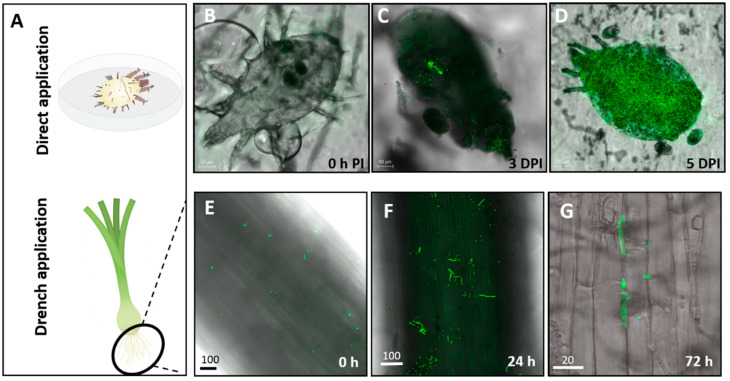
(**A**) Assessment of Mb7 effect on *Rhizoglyphus robini* mite survival in vitro in Petri dish and in-planta on onion bulblets. (**B**-**D**) Progression of Mb7 on adult *R. robini* mites after direct application by spraying of conidia (10^7^ conidia mL^−1^). (**E**–**G**) Progression of root colonization by Mb7 after drench application to bulblets (10^7^ conidia mL^−1^). PI, post inoculation; DPI, days post inoculation.

**Figure 4:**
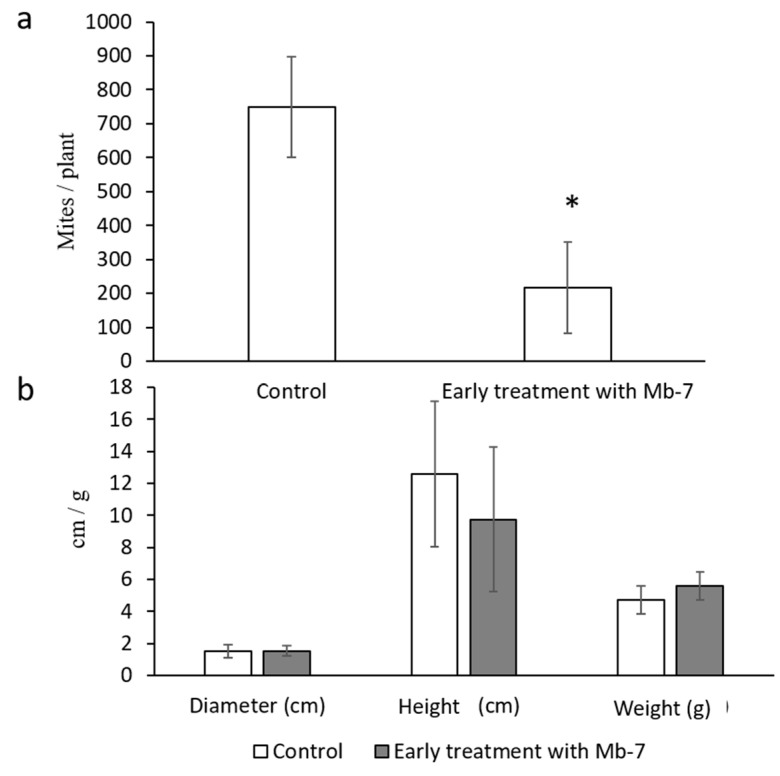
Effect of Mb7 (10^7^ conidia mL^−1^) on adult female *Rhizoglyphus robini* on potted onion plants. (**a**) Number of *R. robini* at 4 weeks after the start of the experiment on control plants and on plants that were treated with Mb7 conidia (* DF = 9, *p* = 0.0128). (**b**) Growth parameters of green onions. Numbers represent bulb diameter (cm), plant height (cm), or fresh plant weight (g). No significant differences were observed in all plant parameters.

In bean leaves and roots (Figure 2A), endophytic colonization was not observed by live imaging, negating the need to search for it in the tissues by re-isolation. The foliar spray resulted in no endophytic colonization in citrus (not shown) or common bean (Figure 2G). Drench application of Mb7 in common bean did not result in visible endophytic root colonization (Figure 2H–J), but in onion, it did, as observed by CLSM in 45 ± 7% of the samples (Figure 3F,G). Mb7 was colonizing the onion tissue by hyphae extending in between the onion cells without penetrating into the cells (Figure 3G). All attempts to recover Mb7 from onion root tissue failed, due to contamination of the culture media by *Fusarium* spp., even though a selective medium was used. No significant differences in plant growth parameters were observed between the onion plants endophytically colonized with Mb7 and the control onion plants (Figure 4B; one-way ANOVA, fresh plant weight: DF = 1, *F* = 0.519, *p* = 0.487; plant height: DF = 1, *F* = 0.19, *p* = 0.66; bulb diameter: DF = 1, *F* = 0.02, *p* = 0.87).

### 3.2. Assessment of the Effect of Directly Applied Mb7 to Mites

**Figure 5:**
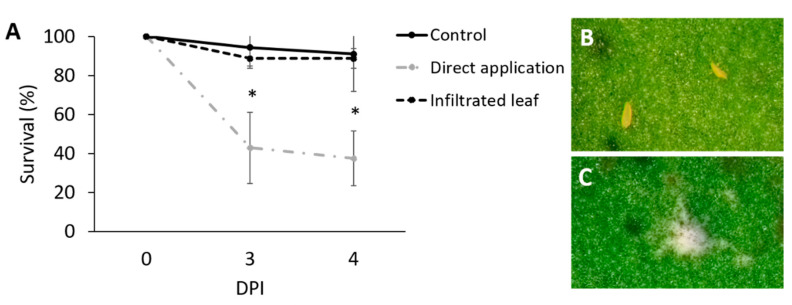
Assessment of Mb7 effect on *Phyllocoptruta oleivora* by leaf disc assay. (**A**) Survival of adult *P. oleivora* after direct exposure to Mb7 conidia (10^7^ conidia mL^−1^) and exposure to infiltrated leaves by 0.1 mL (10^7^ conidia mL^−1^). * Significant difference (DF = 8, *F* = 15.4, *p* < 0.0001). (**B**) Mites in the control group. (**C**) Mite cadaver from the direct-application group showing typical fungal infection. DPI, days post inoculation.

**Figure 6:**
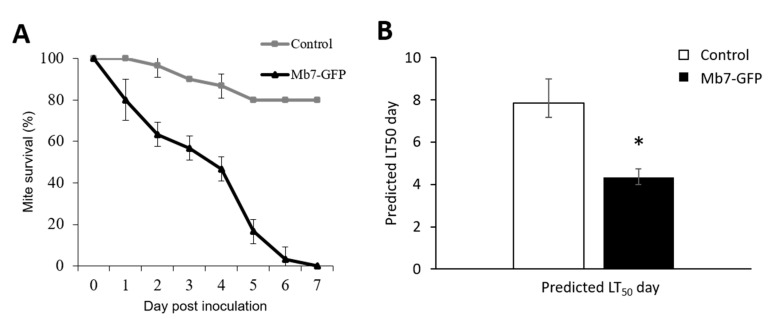
Assessment of Mb7 effect on survival of *Rhizoglyphus robini in vitro*. (**A**) Survival of adult *R. robini* after direct exposure to Mb7 conidia (10^7^ conidia mL^−1^). (**B**) LT_50_ analysis of Mb7 against *R. robini* adult mites (DF = 3, χ^2^= 322.5, *p* < 0.0001). * Significantly different at *p* < 0.05.

**Figure 7:**
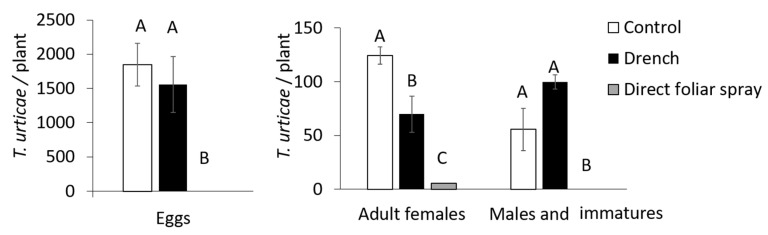
Effect of application of Mb7 (10^7^ conidia mL^−1^) as a drench or foliar spray on number of eggs, adult females, males and immatures of *Tetranychus urticae* two weeks after treatment on bean plants. Different letters above the columns indicate significant difference (Eggs: DF = 2, *F* = 9.87, *p* < 0.005; females: DF = 2, *F* = 34.85, *p* < 0.0001; males and immatures: DF = 2, *F* = 9.02, *p* < 0.005).

Mb7 was pathogenic to *R. robini*, *P. oleivora*, and *T. urticae* when applied directly to the mites as a conidial suspension (Figure 5, Figure 6 and Figure 7, Table 1). The mortality of *P. oleivora* following inoculation with Mb7 was significantly higher than that observed in the control group, with 63% mortality among the treated mites and 8% mortality among mites in control group by 4 DPI (DF = 8, *F* = 15.4, *p* < 0.0001; Figure 5A) and visible mycosis on cadavers in Mb7 treatment and not in control (Figure 5B,C).

The efficacy of Mb7 against *R. robini* females was examined in Petri dishes lined with conidia-inoculated filter paper. This set-up exposed the mites to the conidia continuously and facilitated the daily monitoring of their survival. Mb7 caused significant mortality to adult *R. robini* females, 43% at three days post inoculation (DPI), with a mortality rate of 100% by 7 DPI (*t*-test analysis for 3 DPI: DF = 8, *t* = 2.545, *p* < 0.05; for 7 DPI: DF = 8, *t* = 15.4, *p* < 0.0001; Figure 6A). By 7 DPI 20% mortality was measured in the control group. The calculated LT_50_ for *R. robini* females exposed to Mb7 was 4.3 days and 8 days for mites in the control (DF = 3, χ^2^ = 322.5, *p* < 0.0001; Figure 6B). On the day of exposure to the conidia-treated filter paper (0 h post inoculation, Figure 3B), many conidia were seen on the mites’ bodies. Three days later, Mb7 hyphae were observed inside mite cadavers (Figure 3C) and by 5 DPI, mite cadavers were fully colonized (Figure 3D).

Direct spray application of Mb7 to *T. urticae* resulted in significant reduced numbers of females compared to number of mites in control group (DF = 2, *F* = 34.85, *p* < 0.0001; Figure 7) and no counts of males and immatures together at 14 DPI (DF = 2, *F* = 9.02, *p* < 0.005; Figure 7). In addition, while in control group, an average of 1846 eggs per plant were counted, no eggs were found on plants that had been treated with Mb7 (DF = 2, *F* = 9.87, *p* < 0.005; Figure 7). Mb7 conidia were adhering and germinating on the mites by 3–5 DPI (Figure 2B,C) and they were completely colonized by hyphae at 7 DPI (Figure 2D).

### 3.3. Assessment of the Effect of Mb7, as a Leaf and Root Endophyte, on Mites

Each plant-inoculation technique was chosen to target the mites’ niche and to compare differences in mite survival when directly exposed to conidia (3.2) vs. exposure to plant tissue colonized by Mb7. Citrus leaves that had been infiltrated with Mb7 and in which endophytic colonization was confirmed were exposed to *P. oleivora*. Mite survival on these leaves was 90% and did not differ significantly from that on untreated leaves (Figure 5A; repeated-measures, two-way ANOVA).

Since no endophytic colonization of Mb7 was observed in bean plants after the spray application (Figure 2G), this treatment was not included in our assessment of Mb7 effect as a leaf endophyte on mites. *T. urticae* placed on plants previously sprayed with Mb7 collected conidia on their cuticles, but no signs of infection were observed (Figure 2E,F).

Our finding that 45% of the fungus-treated onion roots were colonized with Mb7 at 3 DPI led us to examine the effects of enriching the complex onion bulb tissues with Mb7. An early drenching of onion bulblets with Mb7 before mite were released to the plants significantly reduced bulb mite numbers by 70% compared with control over a four-week period (Figure 4A; one-way ANOVA, DF = 9, *F* = 7.127, *p* = 0.0128).

### 3.4. Assessment of the Effect of Mb7, as a Rhizosphere Resident, on Mites

Drench applications of Mb7 to bean plants resulted in rhizosphere enrichment with Mb7, and the fungus became established there (Figure 2G–J). Fourteen days after *T. urticae* females were released onto the foliage of rhizosphere-enriched bean plants, the numbers of males, immatures and eggs were not significantly lower than those observed on untreated control plants, in contrast with the significant reduction observed following the foliar spray application of Mb7 (Figure 7; one-way ANOVA, males and immatures: DF = 2, *F* = 9.02, *p* < 0.005; eggs: DF = 2, *F* = 9.87, *p* < 0.005). However, the number of females on the drench-inoculated plants was significantly reduced by 56% compared to control (Figure 7; one-way ANOVA, DF = 2, *F* = 34.85, *p* < 0.0001).

## 4. Discussion

Here, we assessed the interactions of Mb7 with three phytophagous mites in three different plant systems including a monocot and two dicots. The plant colonization by Mb7 was evaluated by CLSM [29] and plant tissue recovery [30]. We observed differences in results obtained using the classical method of evaluating endophytic colonization by re-isolation i.e., confirmation by tissue recovery and the actual endophytic colonization rates confirmed by live, direct imaging of plant tissue by CLSM. The latter proved to be a reliable tool for monitoring the early stages of Mb7 interactions with plant tissue (as an endophyte or as a rhizosphere resident) and for characterizing the local and systemic colonization of plant tissues. Visualization of fungal entomopathogens expressing GFP was claimed as a valuable methodology with great impact on endophytism study, which still relies on cultural methods (based on the review by Vega [19]).

By CLSM we observed endophytic colonization of citrus leaves and onion roots with Mb7, reported here for the first time (based on reviews by Jaber and Ownley, and Bamisile [18,25]). The unsuccessful colonization of bean leaves with Mb7 and the fact that conidia were not observed germinating on the bean leaves could be a result of adverse conditions that do not support development and plant colonization for Mb7 on bean leaves [19]. Another explanation could be incompatibility of the *M. brunneum* strain used in this study with bean leaves, the presence of non-stimulatory compounds on the surface of those leaves [10,33] or presence of trichomes forming a physical barrier between the spores and the plant surface [34]. In a recent study, 10 different strains of *Metarhizium* did not establish endophytic association with bean plant within a 20-day period although rhizosphere colonization was detected for all the 10 strains [35]. The association described in our study, performed on a single strain, cannot indicate whether it is dictated by the plant host or by the fungal strain. Yet it is reasonable that some fungi exhibiting endophytic lifestyle in one plant may not establish in others. This selective inoculation known as specificity in pathogen host relations may be true for beneficial interactions as well.

The Mb7 strain in our study showed high degree of pathogenicity toward the three mite hosts in direct application. Entomopathogenic fungi play a central role in the natural control of mite populations [7,36,37] and in previous studies Mb7 was already confirmed as a pathogen of ticks (Acari: Ixodidae) [29]. This study describes, for the first time, the pathogenicity and efficacy of *M. brunneum*-7 against the citrus rust mite under laboratory conditions and the bulb mite under laboratory and greenhouse conditions. The only entomopathogenic fungi known to naturally attack the citrus rust mite are *Hirsutella thompsonii* [37] and *Meira* spp. [38]. Commercial products based on *H*. *thompsonii* have been used to reduce mite infestations. *Meira* spp., mainly *Meira geulakonigii*, have been identified as plant endophytes that reduce mite populations not by direct colonization, but through the production of toxic metabolites such as argovin [36,39]. In regard to the bulb mite, previous studies have evaluated its susceptibility to entomopathogenic fungi, including *Hirsutella* spp., *Isaria fumosorosea, Metarhizium* spp., and entomopathogenic nematodes [40,41,42]; but only one strain of a *Metarhizium* sp. isolated in Israel was found virulent [42].

*Metarhizium brunneum* soil drenches improved wheat yield and reduced the damage caused by the elaterid, *Limonius californicus* [43]. Furthermore, foliar applications of *M. brunneum* have been shown to lead to transient endophytic colonization and high mortality rates of the moth *Spodoptera littoralis* and the whitefly *Bemisia tabaci* in alfalfa, tomato and melon [44,45]. Based on our results, in which direct application of Mb7, but not endophytic leaf colonization, reduced citrus rust mite populations, we conclude that leaf-endophytic Mb7 most likely does not secrete any acaricidal metabolites, reducing mite numbers only by direct contact of the mites with the conidia and ultimately infection. The results of our study are not in accordance with those of Resquín-Romero et al. [46] and Garrido-Jurado et al. [45], who observed additional mortality of *Spodoptera littoralis* larvae that fed on endophytically colonized plants, as well as insect cadavers and leaf discs in which destruxin A was found. We suggest that the differences between the results of those studies and our findings stem from differences in the experimental system. Further study of the secondary metabolites of Hypocrealean entomopathogenic fungi as endophytes in general, and of *M. brunneum* in particular, may contribute to elucidating the involvement of secondary metabolites in these specific scenarios.

Management of soil pests is not only challenged by a lack of efficient pesticides, but also by a lack of appropriate sampling methods for assessing field populations and predicting outbreaks, a lack of integrated management alternatives, and limited knowledge of pest biology and ecology. The bulb mite is a common soil dweller [47] that attacks onion (*A. cepa*), garlic (*Allium sativum*), lily (*Lilium longiforum*), and ruscus (*Danae racemosa*) in Israel [48,49], and is considered an important pest of these crops worldwide. This mite is not an easy target for chemical pesticides and natural enemies, as it lives in the soil on rotting plant tissue, between bulb scales and inside sprouts. Hence, a management strategy capable of reaching its hidden locations is needed. The results of the current study demonstrate the susceptibility of the bulb mite to Mb7 and the efficacy of drench applications of Mb7 in reducing the bulb mite populations infesting potted onions. We speculate that the mechanisms leading to reduced bulb mite numbers in this scenario include: (i) enrichment of the rhizosphere with Mb7 conidia, which facilitates direct contact of mites with conidia and ultimately infection; (ii) endophytic establishment of Mb7 in the roots, which could engender conidiogenesis, thereby increasing the amount of conidial inoculum; and (iii) establishment of Mb7 in the complex bulb tissues.

In studies conducted on onion bulbs, mites were observed to be attracted to *Fusarium*-infested bulbs and to become established on them, due to the emission of alcohols that attracted them [47,49]. Other studies have shown that this mite can transfer various microorganisms through its alimentary tract [47,50]. Several studies reported antagonistic effect of endophytic entomopathogenic fungi on plants pathogens (review in Vega [19]) and more specifically *M. anisopliae* effectively reduced *Fusarium oxysporum* infection in onion bulbs [51]. The fact that Mb7 was observed developing and endophytically colonizing bulb roots gives rise to questions such as: (i) what are the interactions among the members of the onion rhizosphere microflora?; (ii) are these mites protected from pathogens due to their establishment in niches that are rich in microflora, and as such have developed resistance to pathogens?; and (iii) does the establishment of *M. brunneum* in the rhizosphere impair the establishment of phytopathogenic fungi?

Although we studied a single strain, our observations indicate that Mb7 was readily colonizing endophytically the onion root, a monocot plant, but following drench application to bean root, a dicot plant, Mb7 was detected as a rhizosphere resident with no endophytic colonization of the roots. Similar observations were reported for *Metarhizium* species with a clear preference for monocots than to dicots plants [35]. Our results showed lower abundance of *T. urticae* females on bean plants with Mb7 as a rhizosphere resident, whereas males, immatures, and egg numbers were not affected. As females are the main dispersing life stage of spider mites [52], it is likely that female *T. urticae* were repelled by chemical foliar cues, possibly induced by the Mb7 resident in the rhizosphere. Future studies should address questions regarding plant responses, such as changes in chemical cues; induced resistance and formation of active metabolites, which can effect pests; and the mechanism that enables endophytic establishment of *M. brunneum* in microflora-enriched niches, such as bulbs infested with phytopathogenic microorganisms.

## Figures and Tables

**Table 1:** Plant-host target sites, inoculation techniques, endophytic colonization (average % ± SE), and effects on mite mortality and population reduction (DPI, days post inoculation; WPI, weeks post inoculation). * *Fusarium* spp. Contamination.

Plant Host, Cultivar	Target Pest’s Common Name, Latin Name and Taxonomic Family	Target Sites	Inoculation Technique	Endophytic Colonization, % ± SE		Effect on Mite Pest
				Tissue Recovery	Live Imaging	
*Citrus volkameriana*, Volkamer lemon, ‘Volka’	Citrus rust mite, *Phyllocoptruta oleivora*, Eriophyidae	Leaves Leaves	Foliar spray Leaf infiltration	0 50 ± 12.3%	0 100%	62.6 ± 14.1% mortality 4 DPI 0
*Phaseolus vulgaris*, Common bean, ‘Haricot’	Two-spotted spider mite, *Tetranychus urticae*, Tetranychidae	Leaves Rhizosphere	Foliar spray Drench	— —	0 0	95.3 ± 1–100% reduction 14 DPI 44 ± 16.7% reduction 14 DPI
*Allium cepa*, Spring onion, ‘Ori’	Bulb mite, *Rhizoglyphus robini*, Acaridae	Drench	Rhizosphere	0 *	45 ± 7%	72 ± 10.7% reduction 4 WPI
